# Deciphering the Diagnostic and Natural Therapeutic Implications of Necrosis by Sodium Overload and NK Signatures in Endometriosis Patients

**DOI:** 10.3390/ijms27104535

**Published:** 2026-05-18

**Authors:** Juan Du, Zili Lv

**Affiliations:** School of Medicine and Life Sciences, Chengdu University of Traditional Chinese Medicine, Chengdu 611137, China; apple7863@163.com

**Keywords:** endometriosis, necrosis by sodium overload, natural killer cell, diagnosis, therapeutic agents

## Abstract

Endometriosis (EMT) is characterized by a chronic inflammatory disorder in the female reproductive system, posing significant challenges to global women’s health. Necrosis by Sodium Overload (NESCO) is a novel immunogenic programmed cell death (PCD) pattern that may potentially inhibit natural killer (NK) cell activation by increasing cytotoxicity and the inflammatory response in the EMT microenvironment. By integrating three bulk datasets to compare endometrium tissues between endometriosis patients and normal controls and the NESCO gene list from a public database, we identified NK- and NESCO (NN)-associated hub genes via integrative bioinformatic analyses utilizing Limma, WGCNA, CIBERSORT and machine learning frameworks. The diagnostic performance of NN-associated hub genes was evaluated across the three aforementioned datasets and two independent validation sets. Furthermore, their molecular and immune features were estimated at the bulk and single-cell transcriptomic levels. In addition, endometriosis patients were classified into two novel molecular subgroups based on consensus clustering of NN. Finally, the Traditional Chinese Medicine Systems Pharmacology Database and Analysis Platform (TCMSP) and molecular docking were used to identify compounds in Chinese traditional medicine (CTM) that can target NN-associated hub genes for endometriosis treatment. *FABP4* and *SLC2A1* can be considered NN-associated hub genes that are involved in EMT pathogenesis, and natural compounds including the CTM GuiZhiFuLingWan (GZFLW) can be considered therapeutic agents for EMT treatment as they target *FABP4* and *SLC2A1*. Our study is the first to reveal the diagnostic and druggable roles of NESCO and NK cells, the corresponding molecular and immune features of NN-associated hub genes, and the therapeutic potential of GZFLW.

## 1. Introduction

Endometriosis (EMT), which exerts both direct and indirect influences on reproductive health, is a complex and systemic condition characterized by multiple contributing factors, affecting approximately 10% of women of reproductive age and leading to significant morbidity, including pain and infertility [1]. The pathophysiology of EMT involves complex molecular mechanisms characterized by chronic inflammation and immune dysregulation of the peritoneal immune microenvironment, which lead to the growth of endometrium-like tissues outside the uterine cavity [2]. In addition, chronic inflammation is perpetuated by the presence of ectopic endometrial tissue, which triggers a local immune response, often resulting in an imbalance between pro-inflammatory and anti-inflammatory cytokines [3]. Current treatments for EMT, such as hormonal therapies and surgical interventions, have shown limited efficacy and high recurrence rates, indicating a pressing need for novel therapeutic approaches [4]. Innovative strategies, including targeting specific signaling pathways and developing immunomodulatory therapies, are being explored to enhance treatment outcomes [5]. However, the heterogeneity of the peritoneal immune microenvironment for EMT patients contributes to the limitations of immunological therapy [6].

It is known that immunogenic cell death (ICD) patterns contribute to EMT progression and EMT immune environment heterogeneity [7]. Necrosis by Sodium Overload (NESCO) is a type of ICD caused by sodium influx, which is regulated by transient receptor potential Melastatin (*TRPM4*) [8]. Reports have implicated that the Sodium–Potassium–Chloride Cotransporter axis and voltage-gated sodium channel dysregulations contribute to EMT progression, leading to pain and fertility issues in EMT patients [9,10]. However, NESCO mechanisms in EMT pathogenesis have not yet been further elucidated. Natural killer (NK) cells, as the first line of defense in the innate immune system, can reduce cytotoxic effects in women with EMT [11], and their dysfunction can lead to immune homeostasis dysregulation and infertility in EMT patients [12,13]. From an immunological perspective, NK cells play a dominant role in EMT pathogenesis compared to other immune cells [11]. However, the NESCO mechanisms involved in regulating NK cells, EMT pathogenesis, and immune heterogeneity still remain unclear, and elucidating NESCO mechanisms in NK cells can provide additional insights into EMT pathophysiology and clinical translation.

**Figure 1:**
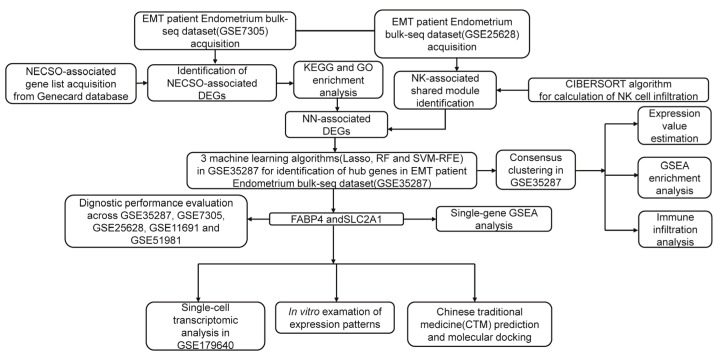
The workflow of this study.

Overall, by integrating integrative bioinformatic analysis and multi-omics data, our study is the first to trace the NESCO mechanisms regulating NK cells to elucidate EMT pathogenesis and immune microenvironment heterogeneity. In addition, we point out that NESCO- and NK activation (NN)-associated molecules, including FABP4 and SLC2A1, can be considered hub genes for EMT diagnostics and therapeutic targeting. Indeed, compounds of the anti-inflammatory Chinese traditional medicine (CTM) GuiZhiFuLingWan (GZFLW) were found to display therapeutic potential for EMT treatment by targeting Solute Carrier Family 2 Member 1 (*SLC2A1*) and Fatty Acid-Binding Protein 4 (*FABP4*), which may change the EMT immune microenvironment, thus alleviating EMT-induced inflammation [14]. This study aimed to investigate the clinical applications and mechanisms of EMT. The workflow of this study is illustrated in Figure 1.

## 2. Results

### 2.1. Identification of NESCO-Associated DEGs in EMT Patients

**Figure 2:**
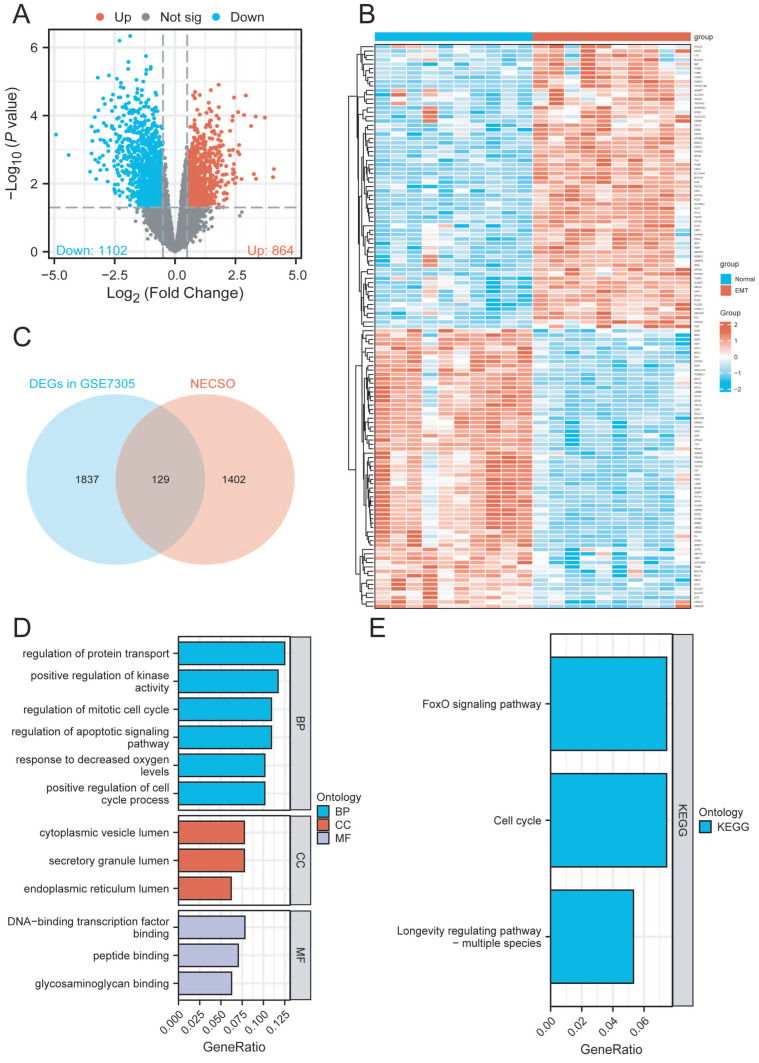
Identification of NESCO-associated DEGs in EMT patients. (**A**) Volcano plot illustrating DEGs in GSE7305. (**B**) Expression patterns of NESCO-associated DEGs in GSE7305 illustrated as a heatmap. (**C**) Venn plot illustrating the acquisition of NESCO-associated DEGs in GSE7305. (**D**,**E**) GO and KEGG enrichment of the molecular and biological functions of NESCO-associated DEGs.

After normalization and standardization of GSE7305, we identified 1966 DEGs in EMT patients, including 864 up-regulated and 1102 down-regulated DEGs (Figure S2B and Figure 2A–C). Next, we intersected these DEGs with the NESCO-associated gene list and then identified 129 NESCO-associated DEGs (Figure 2B,C). Furthermore, their molecular and biological functions were also enriched (Figure 2D,E), and the results indicated that these NESCO-associated DEGs were mainly involved in the cell cycle, transcriptional activity, FoxO signaling pathways, apoptosis, and oxygen-level regulation, with oxygen-level regulation being closely related to programmed cell death (PCD) regulation.

### 2.2. Integrating WGCNA for NN-Associated Shared DEG Identification in EMT Patients

**Figure 3:**
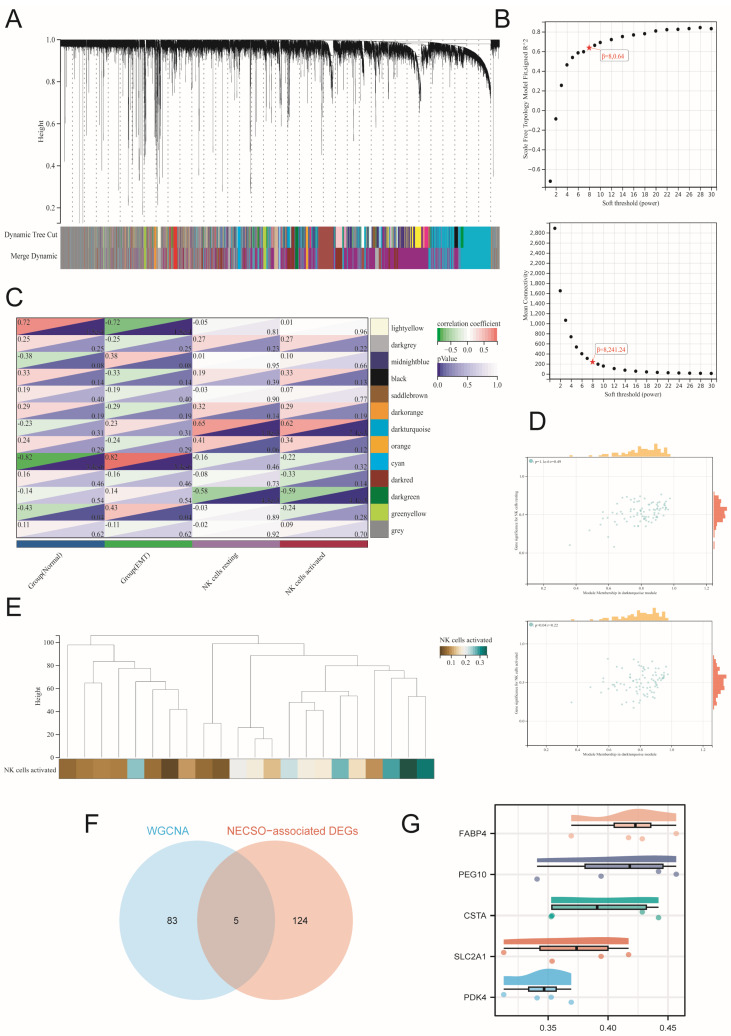
Identification of NN-related shared DEGs in EMT patients. (**A**) Clustering tree of the expression module via WGCNA of GSE25628. (**B**) Scatterplots of representative modules in GSE25628. (**C**) WGCNA-generated heatmap indicating the module–trait relationship in GSE25628. (**D**) Correlated analysis of genes in the dark turquoise module via WGCNA of GSE25628. (**E**) Clustering tree illustrating the relationship between samples and NK cell activation. (**F**) Acquisition of NN-associated DEGs. (**G**) Friend analysis of NN-associated DEGs to assess their importance at the genomic level.

In EMT bulk profile GSE25628, we performed CIBERSORT and WGCNA to identify the model with co-expressed and highly correlated NK cell activation. With a soft power threshold of approximately eight, the dark turquoise module had the highest correlation with NK cell activation (correlation coefficient = 0.65 with *p* = 1.0 × 10^−3^ for resting NK cells and 0.62 correlation coefficient = 0.65 and *p* = 2.1 × 10^−3^ for activated NK cells) (Figure 3A–C,E). Next, we extracted hub genes in the dark turquoise module and identified 88 shared DEGs in GSE25628 (Figure 3D). We then intersected these 88 hub genes with 129 NESCO-associated DEGs, yielding five NN-related DEGs (Figure 3F). Furthermore, we also assessed the importance of five hub genes, and the results showed that *FABP4*, *PEG10*, and *CSTA* displayed the highest importance (Figure 3G). Thus, these preliminary results revealed the co-expression patterns of NESCO and NK cells.

### 2.3. NN-Associated Hub Gene Identification for EMT Patients

**Figure 4:**
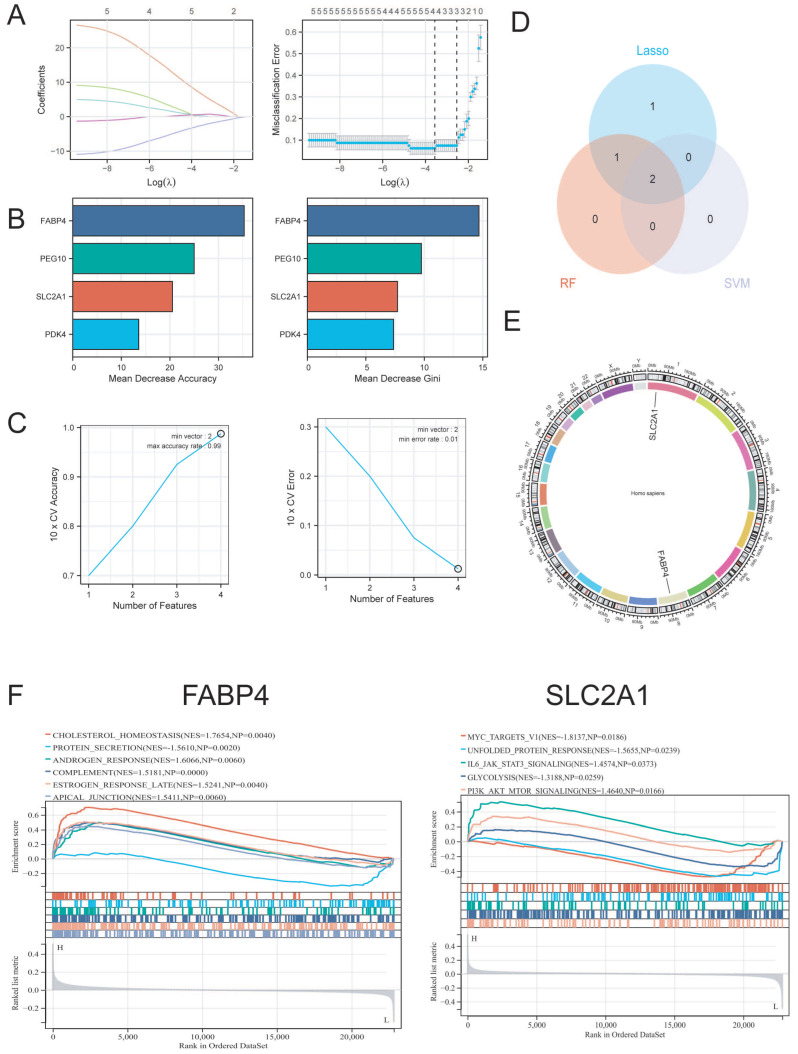
Identification of NN-associated hub genes in EMT patients. (**A**) Lasso logistic regression for the identification of NN-associated hub genes in GSE35287. (**B**) Random forest analysis for the identification of NN-associated hub genes in GSE35287. (**C**) SVM-RFE analysis for the identification of NN-associated hub genes in GSE35287. (**D**) Integration of RF, Lasso logistic regression, and SVM-RFE results to select the optimal NN-associated hub genes in GSE35287. (**E**) Chromatin localization analysis of *SLC2A1* and *FABP4*. (**F**) Single-gene GSEA of *SLC2A1* and *FABP4* to identify the molecular functions involved in EMT pathogenesis in GSE35287.

Using the EMT bulk training set GSE35287, we performed integrative Lasso logistic regression, RF and SVM-RFE to identify NN-associated hub genes in EMT patients, yielding SLC2A1 and FABP4 (Figure 4A,E). In addition, we performed single-gene GSEA of SLC2A1 and FABP4 in GSE35287 to gain insights into the molecular features of these two molecules in EMT pathogenesis (Figure 4F). The results showed that these molecules mainly regulate cholesterol metabolism, the unfolded protein response, IL6-JAK-STAT3 pathways, and PI3K-AKT-mTOR signaling pathways, which were highly associated with EMT pathogenesis and immune dysregulation [15,16,17] (Figure 4F). These results indicated that two NN-associated hub genes, namely *SLC2A1* and *FABP4*, were closely related to EMT pathophysiology.

### 2.4. Diagnostic Model Construction of NN-Associated Hub Genes in EMT Patients

**Figure 5:**
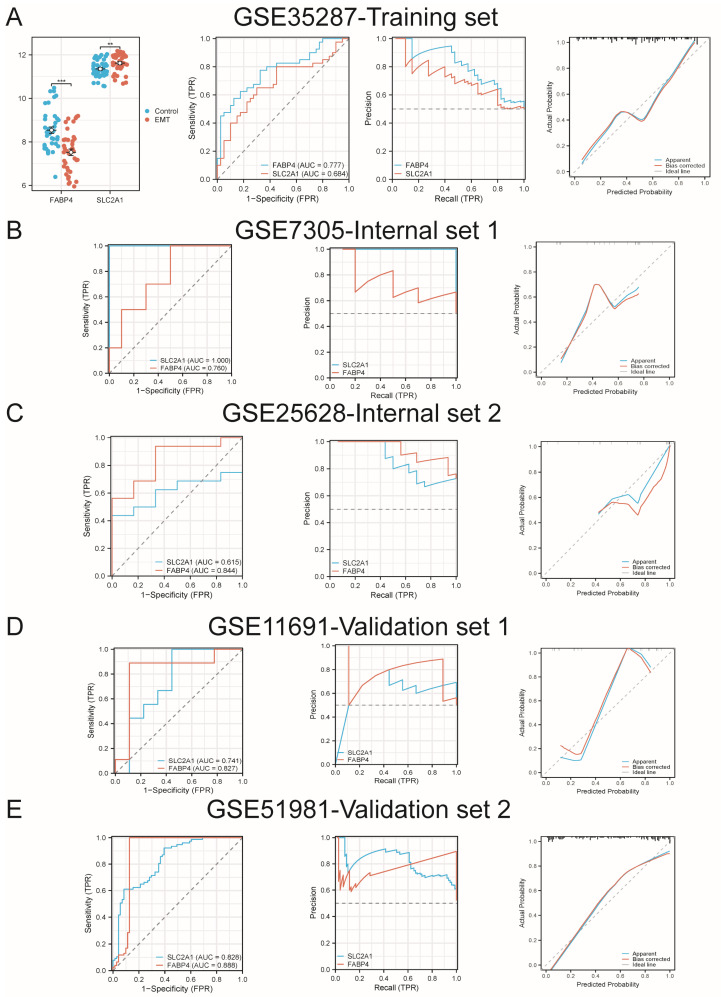
(**A**) Expression and diagnostic performance of *FABP4* and *SLC2A1* for EMT evaluation in patients. (**B**) The diagnostic performance of *FABP4* and *SLC2A1* in GSE7305 is shown using an ROC curve, a PR curve, and calibration analysis. (**C**) The diagnostic performance of *FABP4* and *SLC2A1* in GSE25628 is illustrated using an ROC curve, a PR curve, and calibration analysis. (**D**) The diagnostic performance of *FABP4* and *SLC2A1* in GSE11691 is visualized with an ROC curve, a PR curve, and calibration analysis. (**E**) The diagnostic performance of *FABP4* and *SLC2A1* in GSE51981 is illustrated using an ROC curve, a PR curve and calibration analysis. ** *p* < 0.01, *** *p* < 0.001.

In EMT bulk training set GSE35287, a down-regulated expression pattern was observed for *FABP4*, while *SLC2A1* displayed an up-regulated expression pattern (Figure 5A). In addition, combined ROC, PR, and calibration analyses of GSE35287, GSE7305, GSE25628, GSE11691 (EMT bulk independent validation set), and GSE51981 (EMT bulk independent validation set) indicated that *FABP4* and *SLC2A1* are potential powerful diagnostic biomarkers for EMT onset with favorable accuracy and efficacy (Figure 5B–E). These results also highlighted the role of NECSO and NK cell integration in EMT prediction.

### 2.5. NN-Associated Molecular Subgroup Identification in EMT Patients

**Figure 6:**
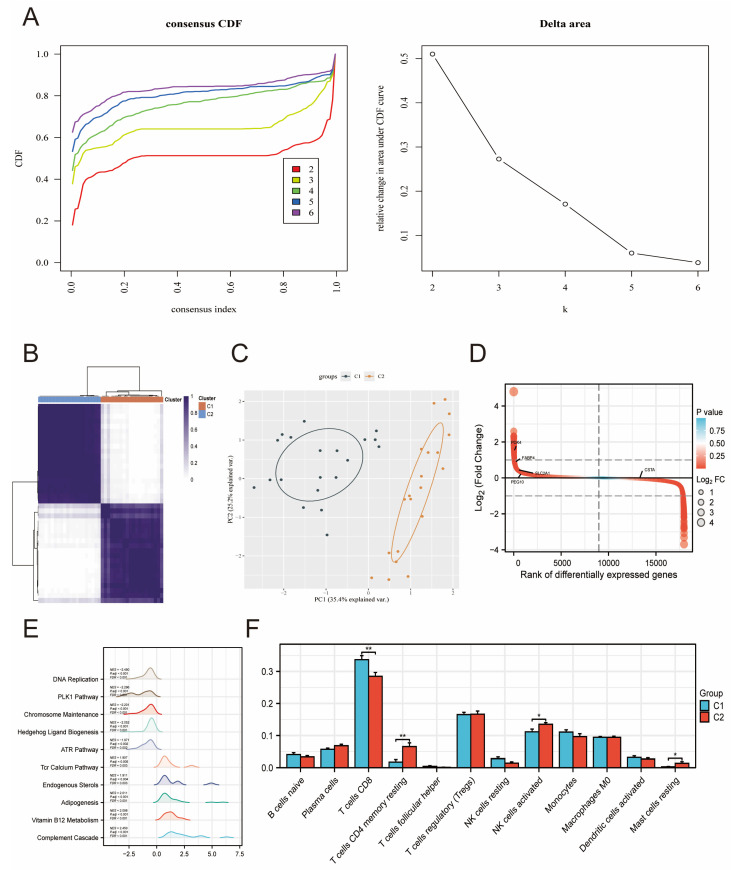
NN-associated molecular subgroup identification in EMT patients. (**A**,**B**) CDF curve of consensus clustering results. (**C**) PCA illustrating the separation between C1 and C2. (**D**) Expression patterns of NN-associated DEGs between C1 and C2. (**E**) GSEA revealed functional differences between C1 and C2. (**F**) Immune infiltration differences between C1 and C2. * *p* < 0.05, ** *p* < 0.01.

Risk stratification can facilitate precision and personalized medicine for EMT patients. In this study, we performed consensus clustering in GSE35287 based on five NN-associated DEGs. The results indicated that these NN-associated DEGs could be used to divide EMT patients into two molecular subgroups (C1 and C2) with clear separation (Figure 6A–C). In addition, the expression patterns of NN-associated DEGs were compared between C1 and C2, with *PDK4*, *FABP4*, *SLC2A1*, and *PEG10* showing higher expression patterns in C1 compared to C2 and CSTA showing the opposite trend (Figure 6D). The molecular and immune feature differences between C1 and C2 were also estimated (Figure 6E,F). The results showed that there were differences in metabolic and immune pathways between C1 and C2 (Figure 6E). In addition, the proportions of CD8+ T cells, inactivated CD4+ T cells, and activated NK cells differed between C1 and C2 (Figure 6F).

### 2.6. Performance Evaluation of NN-Associated Hub Genes at the Single-Cell Transcriptomic Level in EMT Patients

**Figure 7:**
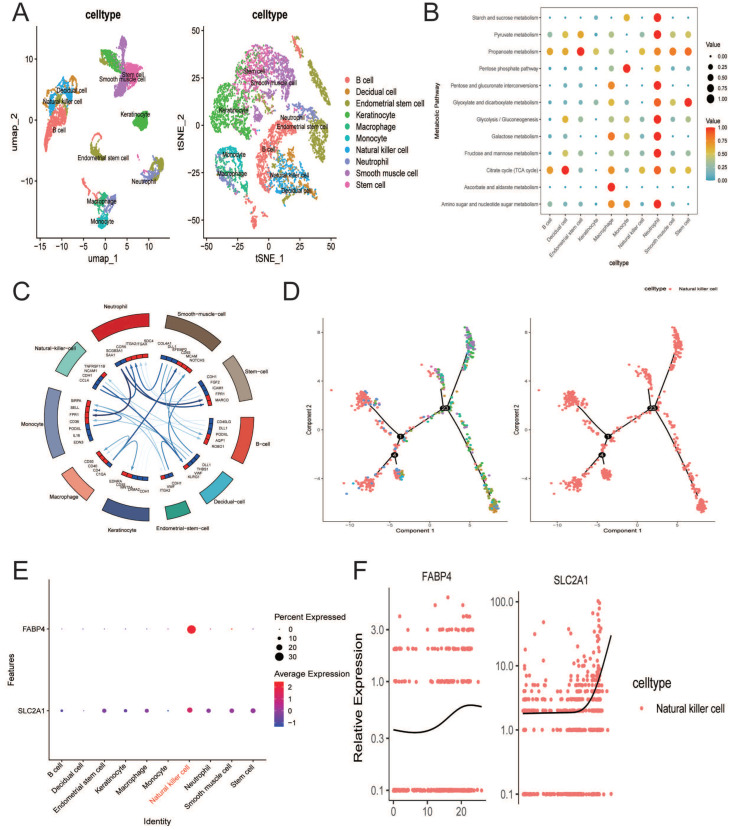
Single-cell transcriptomic analysis of SLC2A1 and FABP4 in EMT patients. (**A**) Cell annotation analysis. (**B**) Metabolic heterogeneity among various cell types in EMT patients. (**C**) Cell chat patterns among various cell types in EMT patients. (**D**) Pseudo-time trajectory analysis of NK cells at the EMT single-cell level. (**E**) Expression patterns of SLC2A1 and FABP4 in 10 distinct cell types. (**F**) Spatio-temporal expression patterns of SLC2A1 and FABP4 in NK cells. Colors indicate annotated cell types or pseudo-time states. Arrows and lines indicate the inferred pseudo-time trajectory or cell–cell communication patterns.

The single-cell RNA sequencing (scRNA-seq) data employed in this study were obtained from the GSE179640 dataset, which includes nine EMT patient-derived tissue samples. Initially, the dataset underwent normalization and QC measures (Figure S1A,B). To facilitate more precise identification of cell types, we classified the cells into 24 distinct clusters based on the expression profiles of well-established markers (Figure S1C,D). Subsequently, we identified the presence of 10 unique cell types, namely B cells, decidual cells, endometrial stem cells, keratinocytes, macrophages, monocytes, natural killer cells, neutrophils, smooth muscle cells, and stem cells (Figure 7A and Figure S1E). Moreover, we assessed energy metabolism patterns and cell communication dynamics across these 10 cell types, with a particular focus on NK cells (Figure 7B,C). The results showed that the TCA cycle and propanoate metabolism signals were relatively amplified compared to other metabolic signals in NK cells (Figure 7B). In addition, NK cells were found to mainly communicate with neutrophils and decidual cells via *CCL4-CCR5* and *CDH1-KLRG1* signals (Figure 7C). Furthermore, we found that *SLC2A1* and *FABP4* were mainly expressed in NK cells (Figure 7E). Finally, the differentiation pattern of NK cells and the spatio-temporal expression patterns of *FABP4* with *SLC2A1* in NK cells were also estimated (Figure 7D,F). The results indicated that the number of NK cell number increased in the terminally differentiated stage, and *FABP4* and *SLC2A1* showed higher expression in NK cells at the terminally differentiated stage (Figure 7D,F).

### 2.7. In Vitro Evaluation of the Expression of NN-Associated Hub Genes and Potential Therapeutic Agent Enrichment in EMT Patients

**Figure 8:**
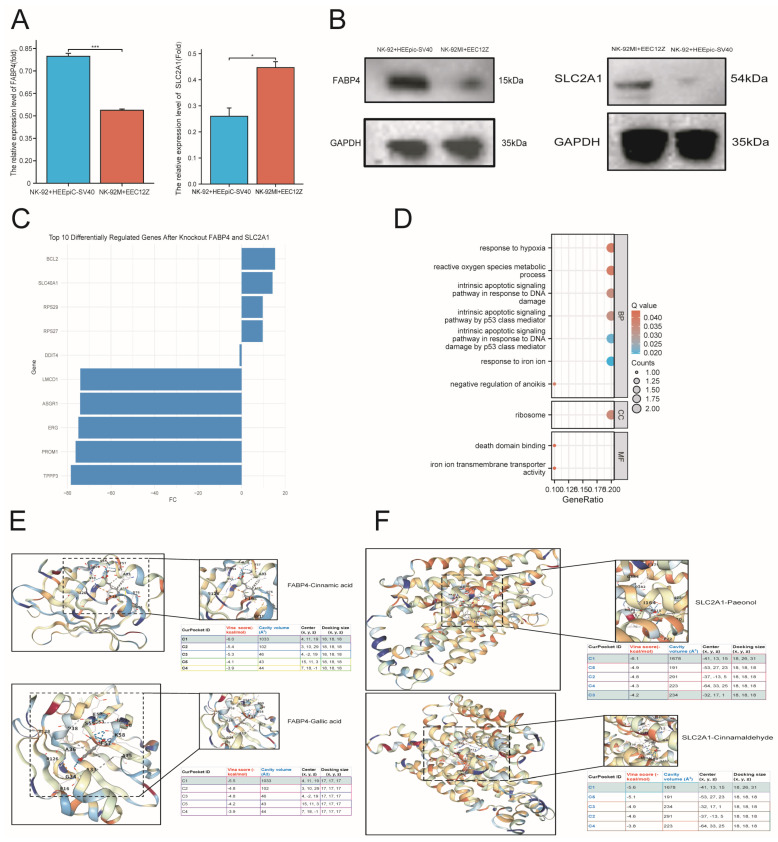
In vitro examination of *FABP4* and *SLC2A1* expression patterns and corresponding therapeutic agent enrichment. (**A**,**B**) In vitro expression of FABP4 and SLC2A1 in co-cultured NK-92MI with EEC12Z cells compared to that in NK-92 cells cultured with HEEpiC-SV40 cells at the mRNA and protein levels. (**C**,**D**) NK virtual cell KO of FABP4 and SLC2A1 for the investigation of their mechanisms of action in NK cells. (**E**,**F**) Therapeutic agent enrichment targeting SLC2A1 and FABP4 for EMT treatment. Colors indicate different experimental groups, enriched terms, or compound–target categories, as shown in the corresponding panel legends. * *p* < 0.05, *** *p* < 0.001.

*FABP4* was down-regulated in co-cultured NK-92MI with EEC12Z cells compared to NK-92 cells cultured with HEEpiC-SV40 cells at the mRNA and protein levels (Figure 8A,B). However, *SLC2A1* showed the opposite trend (Figure 8A,B). To determine the function of *FABP4* and *SLC2A1* in NK cells, we performed NK virtual cell KO of both genes in NK cells. We established the top 10 differentially expressed molecules after *FABP4* and *SLC2A1* KO and discovered that these molecules were mainly involved in PCD and ferroptosis, highlighting their potential role for regulating NESCO in NK cells (Figure 8C,D). Additionally, GZFLW includes the GuiZhi, FuLing, TaoRen, DanPi, and ShaoYao formulas, and after screening for compound–target interactions, we confirmed that Paeonol and cinnamaldehyde could be considered as ideal compounds for EMT treatment as they both target *SLC2A1* (Figure 8E). Additionally, cinnamic acid and gallic acid could also be considered as ideal compounds for EMT treatment as they target *FABP4* (Figure 8F).

## 3. Discussion

EMT is a complex and debilitating condition that affects a significant number of women worldwide, often leading to chronic pain and infertility [18]. Current treatment options are limited and frequently involve invasive procedures or hormonal therapies that may not address the underlying pathophysiology [18]. This highlights the necessity for novel diagnostic and therapeutic strategies. In this study, the integrative bioinformatic and machine learning pipelines highlighted the predictive potential of *SLC2A1* and *FABP4* for EMT onset. In addition, the in vitro study and in silico enrichment analysis highlighted the potential role of *SLC2A1* and *FABP4* in regulating NESCO in NK cells involved in EMT pathogenesis. In addition, the therapeutic potential of GZFLW against EMT was determined via network pharmacology and molecular docking analyses. Taken together, our study is the first to decipher the role of NN and GZFLW in EMT prediction and treatment.

*FABP4* is known for its involvement in lipid metabolism and inflammatory responses, and its elevated expression has been linked to chronic inflammatory diseases [19]. In addition, down-regulated expression of FABP4 can lead to the impairment of NK cell maturation in lung adenocarcinoma (LUAD) [20]. In addition, FABP4 can be considered a driver of PCD pathways, such as apoptosis [21]. *SLC2A1*, which encodes a glucose transporter, is essential for cellular glucose uptake and metabolism [22], and a report pointed out that *SLC2A1* can be considered a therapeutic target as it mediates the interplay between ferroptosis and immune infiltration in EMT [23]. Indeed, GZFLW, as a CTM, has been reported to be effective against polycystic ovary syndrome (PCOS) as it can attenuate oxidative stress and inflammation, which are related to the phosphatidylinositol 3-kinase (*PI3K*)/protein kinase B (*AKT*)/nuclear factor kappa-light-chain-enhancer of activated B cells (*NF-κB*) and nuclear factor erythroid 2-related factor 2 (*Nrf2*)/heme oxygenase-1 (*HO-1*) pathways [24]. However, the link between *FABP4*, *SLC2A1*, and EMT pathogenesis remains unclear. In the present study, we illustrated that *FABP4* and *SLC2A1* can potentially regulate various signaling pathways and PCD patterns in NK cells in EMT patients, highlighting their important roles for regulating NK cells in the EMT immune microenvironment. In addition, we also elucidated their differentially expressed trends in cells co-cultured in vitro, highlighting their potential for regulating the NK–epithelial axis in EMT pathogenesis. Furthermore, the *FABP4* and *SLC2A1* KO experiments in NK virtual cells indicated their roles in PCD regulation and as ion transporters. Significantly, after network pharmacology and molecular docking validation, we also discovered the potential therapeutic effect of anti-inflammatory CTM-GZFLW in EMT treatment [25].

## 4. Materials and Methods

### 4.1. Data Source

All bulk datasets were downloaded from the gene expression omnibus (GEO) database (https://www.ncbi.nlm.nih.gov/geo/, accessed on 10 April 2026) [26]. These datasets, including GSE7305, GSE25628, GSE35287, GSE51981 and GSE11691, were collected for comparison of endometrium samples with normal controls and were acquired via the GEOquery package (version 2.78.0) in R [27]. GSE7305 was based on GPL570, which includes 10 normal samples and 10 EMT patient samples. GSE25628 was based on GPL571, which includes 6 normal samples and 16 EMT patient samples. GSE35287 was based on GPL6244, which includes 40 normal samples and 40 EMT patient samples. GSE51981 was based on GPL570, which includes 71 normal samples and 77 EMT patient samples. In addition, we downloaded a single-cell transcriptomic dataset (GSE179640) that includes 9 ectopic endometrium patient samples from the GEO database. The NESCO gene list with a threshold greater than 1 was downloaded from the Genecard database, as illustrated in Table S1. All bulk profiles were normalized using the Limma package (version 3.60.0) in R, and results are shown in Figure S2 [28].

### 4.2. Identification of DEGs

Adhering to the criteria of an adjusted *p*-value of less than 0.05 and an absolute log fold change (FC) greater than 0.5, the Limma package in R software was utilized to normalize and identify differentially expressed genes (DEGs) within the dataset GSE7305 [28]. Following this, the identified DEGs were cross-referenced with gene lists associated with NESCO to extract NESCO-associated DEGs. The results of this intersection were graphically represented using Venn diagrams. Additionally, the visualization of DEGs was accomplished by creating heatmaps and volcano and heatmap plots using the ggplot2 (version 3.5.1) and complexheatmap packages (version 2.20.0) in R [29,30]. To gain deeper insights into the potential biological functions of the target genes, a functional enrichment analysis was performed. The Gene Ontology (GO) framework is widely used for annotating gene functions, which can be categorized into three distinct areas: Molecular Function (MF), Biological Process (BP), and Cellular Component (CC). Furthermore, Kyoto Encyclopedia of Genes and Genomes (KEGG) enrichment analysis is recognized as a valuable method for investigating gene activities and their corresponding high-level genomic pathways. To achieve a more thorough understanding of the NESCO-associated DEGs, the ClusterProfiler package (version 4.18.4) in R was employed to conduct GO term analysis and to pinpoint significantly enriched KEGG pathways [31].

### 4.3. WGCNA

CIBERSORT utilizes a deconvolution algorithm designed to evaluate the composition and abundance of immune cell types within a cellular mixture by analyzing transcriptomic data. In this study, we initially examined the relative proportions of 22 distinct immune cell types in normal and EMT samples sourced from GSE25628 using the CIBERSORT algorithm [32].

Weighted Gene Co-expression Network Analysis (WGCNA) is employed to detect the gene modules exhibiting high correlations, elucidate the interrelationships among these modules, and examine their associations with external sample characteristics, thereby facilitating the identification of potential biomarkers or therapeutic targets. In our study, WGCNA was implemented using the R package WGCNA to pinpoint modules that exhibit significant relevance to NK cells in EMT patients [33]. Initially, we preprocessed the sample data, thereby eliminating any outliers. Following this, a correlation matrix was generated using the WGCNA software package (version 1.74). The optimal soft threshold was determined to transform the correlation matrix into an adjacency matrix, thus allowing a topological overlap matrix (TOM) to be derived. Subsequently, the TOM-based phase dissimilarity metric was applied to group genes with analogous expression patterns into distinct gene modules through average linkage hierarchical clustering. The modules demonstrating the strongest association with NK cell activation were designated as primary modules for further analysis. After extracting modules with shared NK cell activation, we apply them to NESCO-associated DEGs to acquire NN-associated DEGs. Friend analysis was conducted based on NN-associated DEGs via the GOSemSim package (version 2.32.0) in R.

### 4.4. Machine Learning Algorithms and Diagnostic Model Construction

The Least Absolute Shrinkage and Selection Operator (LASSO) logistic regression analysis represents a data mining technique that utilizes the L1 penalty (lambda) to assign zero coefficients to less significant variables, thereby facilitating the identification of crucial variables and the development of an optimal classification model [34]. The Support Vector Machine–Recursive Feature Elimination (SVM-RFE) analysis serves as a supervised machine learning approach aimed at pinpointing the most relevant core genes by eliminating feature vectors produced by the SVM [35]. The Random Forest (RF) analysis, which is grounded in decision tree methodology, emphasizes the assessment of variable significance by calculating each variable’s importance score [36]. In conjunction with these 3 machine learning techniques, we identified hub genes based on NN-associated DEGs in GSE35287. In addition, single-gene gene set enrichment analysis (GSEA) of hub genes was based on GSE35287, in accordance with the hallmark gene set downloaded from the MSIGDB database. Next, the diagnostic performance of hub genes was assessed in GSE7305, GSE25628, GSE35287, GSE11691, and GSE51981 via receiver operating characteristic (ROC), precision–recall (PR), and calibration analyses using the pROC (version 1.18.5), rms (version 6.7-1), and resourceselection packages (version 0.3.6) in R, respectively.

### 4.5. Single-Cell Transcriptomic Analysis

To commence our investigation, we obtained the single-cell transcriptomic dataset pertinent to EMT, specifically GSE179640, from the GEO database. The analysis of the single-cell RNA sequencing (scRNA-seq) data encompassed several critical phases, including quality control (QC), dimensionality reduction, and marker identification, which were all executed with the Seurat R package (version 5.4.0) [37]. Quality control (QC) was rigorously applied to each cell, adhering to established criteria that required gene counts to fall between 200 and 6000, the unique molecular identifier (UMI) count to exceed 1000, and the mitochondrial gene percentage to remain below 10%. Following the implementation of these QC measures, the data were normalized, facilitating the identification of 2000 genes exhibiting notable variability for subsequent analysis. Post-normalization, dimensionality reduction techniques, particularly t-distributed stochastic neighbor embedding (t-SNE) and uniform manifold approximation and projection (UMAP), were employed [38]. Cell-type annotations were performed using the scMayoMap algorithm available in R software [39], and the expression levels of the target genes were assessed across the various annotated cell populations. Intercellular communication networks were inferred using the CellChat package (version 2.1.2) in R [40]. Additionally, we explored energy metabolic pathways at the single-cell level among the annotated cell populations using the scMetabolism package (version 0.2.1) in R [41]. Notably, pseudo-time analysis of the spatio-temporal expression of the targeted genes within specific cell types was conducted using the monocle2 package (version 2.38.0) in R [42].

### 4.6. Consensus Clustering

The ConsensusClusterPlus package (version 1.68.0) in R was utilized to categorize patients with EMT from the GSE35287 cohort into specific molecular subtypes [43]. To ascertain the most suitable number of clustering partitions, we assessed the relative variation in the area under the cumulative distribution function (CDF) curve across a spectrum of cluster values. This clustering methodology was executed multiple times, ultimately revealing two distinct subgroups that were designated as C1 and C2. An analysis of immune infiltration between the C1 and C2 subgroups was conducted using the CIBERSORT algorithm. Furthermore, a molecular expression analysis of the hallmark gene set obtained from the MSIGDB database was carried out to identify variation in NN-associated DEGs between these subgroups, providing deeper insights into the biological diversity among the molecular subtypes through GSEA.

### 4.7. CTM Evaluation and Molecular Docking

The Traditional Chinese Medicine Systems Pharmacology Database And Analysis Platform (TCMSP) was built based on the framework of systems pharmacology for herbal medicines [44]. To gain insights into the potential of GZFLW for EMT treatment, we performed network pharmacology on the TCMSP database. For pharmacokinetics and component screening of GZFLW, information on its absorption, distribution metabolism, and excretion (ADME) characteristics, such as oral bioavailability (OB), drug similarity (DL), Caco-2 permeability (Caco-2), and blood–brain barrier (BBB) issues, was considered to filter out druggable targets with thresholds of DL ≥ 0.18 and OB ≥ 30% [45]. Next, we also enriched the compound–target interaction network to identify the corresponding compounds targeting hub variables, and molecular docking analysis was performed to assess the interactions between drugs and proteins. The Protein Data Bank (PDB) files for the target proteins were sourced from the RCSB PDB, while the ligand structures were retrieved as SDF files from the Pubchem database. Subsequently, molecular docking was executed to estimate the binding affinities between the selected proteins and the compounds. Initially, PyMOL software (Version 2.6.0) was utilized to eliminate water molecules and ligands, preserving only the protein backbone. Following this, the AutoDock Vina Tool (Version 4.2.6) was employed to identify potential binding sites on the protein surface and to conduct flexible molecular docking. This process allowed the docking scores and binding affinities (Vina scores in kcal/mol) for each identified binding site to be calculated, with the top five sites ranked based on binding energy. The binding site with the lowest binding energy was selected for visualization in PyMOL, which displayed the positions of hydrogen bonds associated with ligand binding in the resulting images, thereby allowing the binding modes and hydrogen bonding interactions to be elucidated.

### 4.8. Cell Lines and Culture

The NK-92MI (ID:SNL-195) and NK-92 (ID: SNL-405) cell lines were purchased from the SUNNCELL company (Wuhan, China), which is included in the NCACC of the Chinese Academy of Sciences. NK-92MI can stably secret IL-2, which can simulate NK-induced inflammation. The HEEpiC-SV40 and EEC12Z cell lines were obtained from the Shanghai Academy of Biological Sciences, which is located in Shanghai, China. HEEpiC-SV40 can be considered a normal uterine cell line, and EEC12Z was established from cells obtained from light red peritoneal lesions of women with EMT. The HEEpiC-SV40 and NK-92 cell lines were co-cultured at the Roswell Park Memorial Institute (RPMI) in the 1640 complete medium at a 1:1 ratio, which was supplemented with a 1% antibiotic mixture consisting of penicillin–streptomycin and 10% fetal bovine serum (FBS, Gibco, Shanghai, China). In addition, the NK-92MI and EEC12Z cell lines were co-cultured at the Roswell Park Memorial Institute (RPMI) under the same conditions. The co-cultures were divided into 2 groups, with the co-cultured HEEpiC-SV40 and NK-92 cell lines as the control group and the co-cultured NK-92MI and EEC12Z cell lines as the EMT group.

#### RNA Extraction and qPCR

Total RNA extraction was performed on the co-cultured NK-92MI and EEC12Z cell lines and the co-cultured HEEpiC-SV40 and NK-92 cell lines using the TRIzol reagent (TaKaRa, Beijing, China), followed by a thorough evaluation of their concentration, purity, and integrity using a NanoDrop spectrophotometer (Thermo Scientific, Waltham, MA, USA). For the reverse transcription step, 1 µg of total RNA was utilized in combination with the HiScript II Q RT SuperMix for qPCR, which comprises a gDNA wiper and a gDNA eraser (Vazyme, Shanghai, China). The concentration, purity, and integrity of the resultant cDNA were assessed with the aforementioned NanoDrop spectrophotometer, and quantitative reverse transcription polymerase chain reaction (qRT-PCR) was performed using the SYBR Green MasterMix (11203ES50, YEASEN, Shanghai, China) and StepOne Software version 2.3 (Applied Biosystems, Carlsbad, CA, USA) over 40 cycles, with three biological replicates for each sample. Data analysis was conducted using the ∆∆Ct (cycle threshold) method, with expression levels normalized to those of the reference gene, namely GAPDH. The primer sequences employed in the qRT-PCR assays are provided in Table 1.

### 4.9. Western Blotting

Following the application of diverse treatments, the cells were washed with ice-cold phosphate-buffered saline (PBS) (Hyclone, Seattle, WA, USA) and subsequently collected through gentle scraping. Total protein extraction was accomplished by lysing the cells with the radioimmunoprecipitation assay (RIPA) lysis buffer (Beyotime, Shanghai, China), which was supplemented with a combination of phosphatase inhibitors (Beyotime, China) and protease inhibitors (Beyotime, Shanghai, China). The resulting cell lysates underwent centrifugation at 14,000× *g* for 15 min at 4 °C. Following centrifugation, the lysates were denatured for 10 min in a 5× SDS-PAGE loading buffer (Beyotime, Shanghai, China). The proteins were subsequently separated using SDS-PAGE and transferred onto polyvinylidene fluoride (PVDF) membranes (Beyotime, Shanghai, China) for Western blotting analysis. The membranes were then blocked with the NcmBlot blocking buffer (NCM Biotech, Suzhou, China) for 10 min and then incubated with primary antibodies diluted in 5% bovine serum albumin (BSA) (Solarbio, Beijing, China) for 8 h at 4 °C. Following this incubation step, the membranes were exposed to secondary antibodies (ThermoFisher, Waltham, MA, USA) that were diluted in the WB secondary antibody diluent solution (Beyotime, Shanghai, China) at a dilution ratio of 1:1000 for 2 h at room temperature. Protein detection was conducted using an enhanced chemiluminescence (ECL) substrate (Thermo Fisher, Waltham, MA, USA), and the quantification of protein expression was executed by analyzing the band densities of the target proteins using ImageJ software version 1.57, with the analysis based on density values relative to the GAPDH protein. The primary antibodies employed in this investigation included the following: FABP4 (ab92501, dilution rate: 1:1000), SLC2A1 (ab115730, dilution rate: 1/100,000), and GAPDH (ab181602, dilution rate: 1:10,000).

### 4.10. Statistical Analysis

All statistical analyses were performed using R software (version 4.5.0) in conjunction with GraphPad Prism software (version 10). Differences between the two groups were determined using either Student’s *t*-test or the Wilcoxon rank-sum test, depending on the data distribution characteristics. For analyses involving multiple groups, one-way ANOVA was employed, followed by Tukey’s post hoc test for further comparisons. The correlation between gene expression levels and immune cell infiltration was assessed using Spearman correlation analysis, and a two-tailed *p*-value of less than 0.05 was considered indicative of statistical significance.

## 5. Conclusions

In conclusion, our study identified the NESCO regulatory patterns involved in EMT pathogenesis in NK cells via integrative bioinformatic analysis and multi-omics and highlighted *FABP4*’s and *SLC2A1*’s diagnostic value and GZFLW’s therapeutic potentials for EMT treatment. However, there are limitations to our study. For example, although our study is based on various bulk and single-cell profiles of EMT patients, such as GSE7305, GSE25628, GSE35287, GSE51981, GSE11691, and GSE179640, which include hundreds of patients from different centers, the immune and molecular mechanisms of *FABP4* and *SLC2A1* in EMT pathogenesis need to be elucidated in pre-clinical studies and their diagnostic potential validated in larger cohort studies. Additionally, the therapeutic effect of GZFLW on EMT should also be assessed in pre-clinical and clinical studies.

## Figures and Tables

**Table 1:** Primers used in this study.

Gene Name	Primer (5′-3′)
*FABP4*	F: GCCAGGAATTTGACGAAGTCAC R: TTCTGCACATGTACCAGGACAC
*SLC2A1*	F: GATGAAAGAAGAGGGTCGGCAGATG R: CAGCACCACAGCGATGAGGATG
*GAPDH*	F: GAGAAGGCTGGGGCTCATTT R: ATGACGAACATGGGGGCATC

## Data Availability

The bulk and single-cell transcriptomic data of endometriosis used in this study are publicly available from the Gene Expression Omnibus (GEO) database under the accession numbers GSE7305, GSE25628, GSE35287, GSE51981, GSE11691, and GSE179640. The NESCO-associated gene list was retrieved from the Genecard database. The hallmark gene sets for GSEA were downloaded from the MSigDB database. The traditional Chinese medicine-related data and compound information were obtained from the TCMSP database, and the ligand and protein structure files for molecular docking were acquired from the PubChem and RCSB PDB databases, respectively. All original experimental data generated from in vitro cell culture, qRT-PCR, and Western blotting assays in this study are available from the corresponding author upon reasonable request. The R code used for bioinformatic analysis, including differential gene identification, WGCNA, machine learning modeling, consensus clustering, and immune infiltration analysis, can be obtained from the corresponding author upon reasonable request. No additional datasets were generated or analyzed during the current study beyond those mentioned above.

## Supplementary Materials

The following supporting information can be downloaded at: https://www.mdpi.com/article/10.3390/ijms27104535/s1.

## Abbreviations

Abbreviation	Full name
EMT	Endometriosis
NESCO	Necrosis by Sodium Overload
NK	Natural Killer (cell)
NN	NESCO and NK (activation)
PCD	Programmed Cell Death
ICD	Immunogenic Cell Death
CTM	Chinese Traditional Medicine
GZFLW	GuiZhiFuLingWan
TCMSP	Traditional Chinese Medicine Systems Pharmacology Database and Analysis Platform
SLC2A1	Solute Carrier Family 2 Member 1
FABP4	Fatty Acid-Binding Protein 4
DEGs	Differentially Expressed Genes
GO	Gene Ontology
KEGG	Kyoto Encyclopedia of Genes and Genomes
WGCNA	Weighted Gene Co-expression Network Analysis
LASSO	Least Absolute Shrinkage and Selection Operator
SVM-RFE	Support Vector Machine-Recursive Feature Elimination
RF	Random Forest
GSEA	Gene Set Enrichment Analysis
ROC	Receiver Operating Characteristic
PR	Precision-Recall
scRNA-seq	Single-Cell RNA Sequencing
QC	Quality Control
UMAP	Uniform Manifold Approximation and Projection
t-SNE	t-distributed Stochastic Neighbor Embedding
ADME	Absorption, Distribution, Metabolism, Excretion
OB	Oral Bioavailability
DL	Drug Likeness
PDB	Protein Data Bank
qPCR/qRT-PCR	Quantitative (Real-Time) Polymerase Chain Reaction
WB	Western Blotting
GEO	Gene Expression Omnibus
TRPM4	Transient Receptor Potential Melastatin 4
