# Supplementary material for: Deciphering the Diagnostic and Natural Therapeutic Implications of Necrosis by Sodium Overload and NK Signatures in Endometriosis Patients
Source: Int J Mol Sci. 2026 May 18;27(10):4535. doi: 10.3390/ijms27104535 (PMC13208043; doi:10.3390/ijms27104535)
Supplement: Supplementary file 1 [file ijms-27-04535-s001.zip › ijms-4213212-supplementary.pdf]

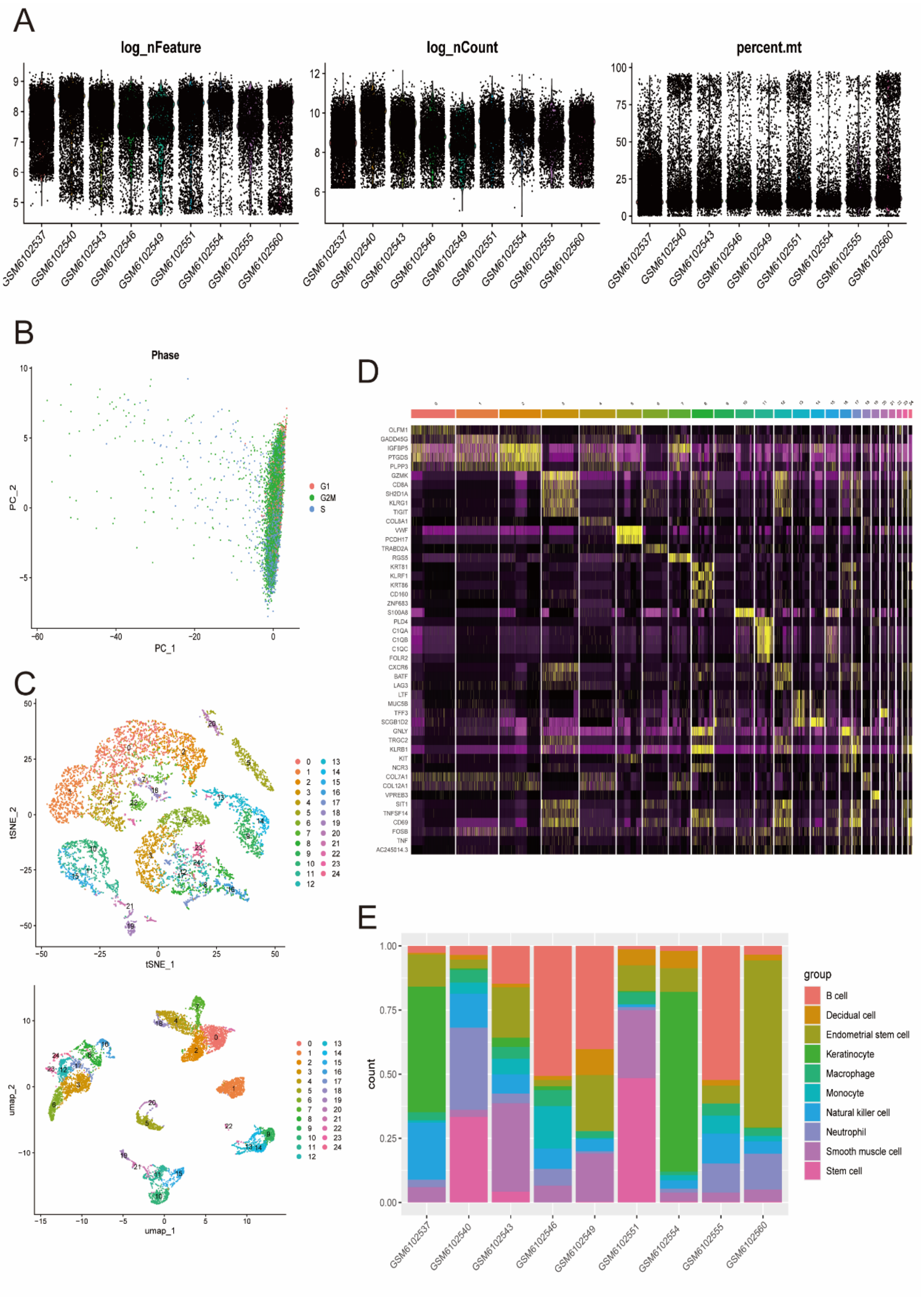

Figure S1: Pre-processing of single-cell data. (A) QC metrics of single-cell data across samples. **(B)** PCA analysis of cells based on cell cycle phase (G1, S, G2M). **(C)** Dimensionality reduction using t-SNE and UMAP, revealing distinct cell clusters. **(D)** Dimensionality reduction using markers. **(E)** Cell proportion among various cell types.

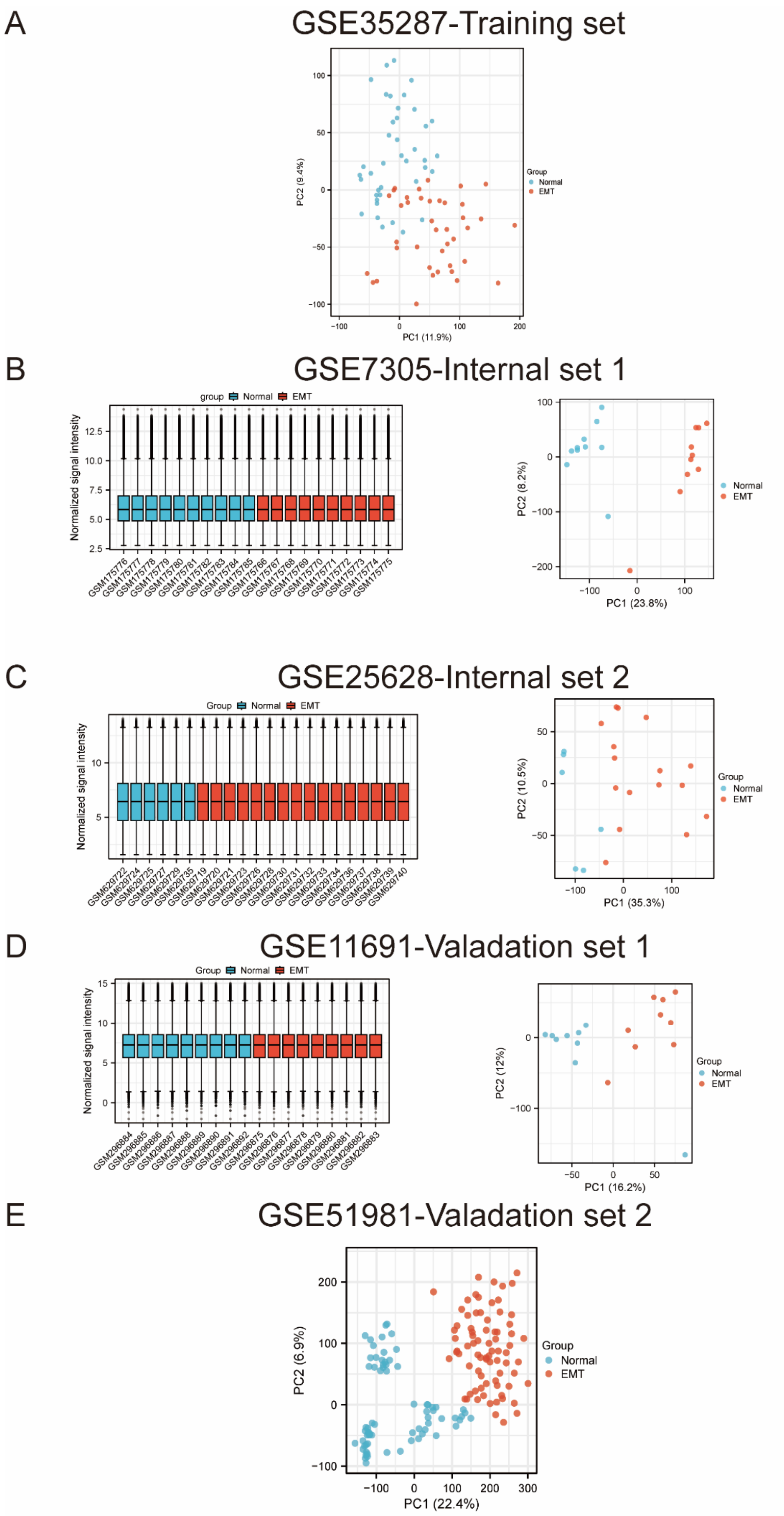
A
GSE35287-Training set
PC2 (9.4%)
PC1 (11.9%)
Group
Normal
EMT
B
GSE7305-Internal set 1
group Normal EMT
Normalized signal intensity
PC2 (8.2%)
PC1 (23.8%)
Normal
EMT
C
GSE25628-Internal set 2
Group Normal EMT
Normalized signal intensity
PC2 (10.5%)
PC1 (35.3%)
Group
Normal
EMT
D
GSE11691-Valadation set 1
Group Normal EMT
Normalized signal intensity
PC2 (12%)
PC1 (16.2%)
Group
Normal
EMT
E
GSE51981-Valadation set 2
PC2 (6.9%)
PC1 (22.4%)
Group
Normal
EMT

Figure S2: Pre-processing of Bulk data.(A) PCA illustration of normalization results of GSE35287.**(B)** Box plot and PCA plot illustration of normalization results of GSE7305.**(C)** Box plot and PCA plot illustration of normalization results of GSE25628. **(D)** Box plot and PCA plot illustration of normalization results of GSE11691. **(E)** PCA plot illustration of normalization results of GSE51981.
